# A Comparative Analysis of MRSA: Epidemiology and Antibiotic Resistance in Greece and Romania

**DOI:** 10.3390/ijms25147535

**Published:** 2024-07-09

**Authors:** Eftychios Vittorakis, Mihaela Laura Vica, Calina Oana Zervaki, Evangelos Vittorakis, Sofia Maraki, Viktoria Eirini Mavromanolaki, Michael Ewald Schürger, Vlad Sever Neculicioiu, Evangelia Papadomanolaki, Lia Monica Junie

**Affiliations:** 1 Department of Microbiology, Iuliu Hatieganu University of Medicine and Pharmacy, 400012 Cluj-Napoca, Romaniamjunie@umfcluj.ro (L.M.J.); 2 Department of Cell and Molecular Biology, Iuliu Hatieganu University of Medicine and Pharmacy, 400012 Cluj-Napoca, Romania; mvica@umfcluj.ro (M.L.V.);; 3 Agios Georgios General Hospital of Chania, 73100 Crete, Greece; 4 Department of Clinical Microbiology and Microbial Pathogenesis, University Hospital of Heraklion, 70013 Crete, Greece; sofiamaraki@gmail.com (S.M.); virnaball@gmail.com (V.E.M.)

**Keywords:** methicillin-resistant *Staphylococcus aureus*, MRSA, epidemiology, molecular characterization, antibiotic resistance, therapeutic perspectives

## Abstract

This study provides a comparative analysis of 243 Methicillin-resistant *Staphylococcus aureus* (MRSA) isolated strains from Greece and Romania, focusing on their epidemiology and antibiotic resistance patterns. Laboratory procedures included phenotypic and automated identification methods, susceptibility testing, DNA isolation, and PCR for detecting antibiotic resistance genes (*MecA*, *SCCmec*). Our study results show significant regional differences. In both regions, males have higher MRSA infection rates than females, but the percentages vary. Greece has a higher incidence of MRSA in younger age groups compared to Romania. The majority of MRSA infections occur in inpatient settings in both countries, highlighting the necessity for enhanced infection control measures. Antibiotic resistance profiles reveal higher resistance to several antibiotics in Greece compared to Romania. A molecular analysis shows a widespread distribution of antibiotic resistance genes among MRSA isolates in Greece. These results highlight the necessity for accomplished preventive strategies and optimized treatment protocols.

## 1. Introduction

Methicillin-resistant *Staphylococcus aureus* (MRSA) poses a significant global health challenge, characterized by its resistance to multiple antibiotics and its ability to cause severe infections in both hospital and community settings. The establishment of the epidemiology and molecular characteristics of MRSA in the BALKAN regions is important for understanding its spread and developing effective therapeutic strategies. MRSA infections present a significant public health challenge in both Greece and Romania, with healthcare-associated and community-associated strains contributing to the burden of disease [1]. Molecular studies utilizing multi-locus sequence typing (MLST) and whole-genome sequencing (WGS) have identified MRSA circulating in healthcare settings and communities. The analysis of genetic determinants highlights the presence of antibiotic resistance genes and mobile genetic elements contributing to MRSA antibiotic resistance [2,3].

MRSA poses a significant challenge to public health worldwide due to its ability to cause a wide range of infections, including skin and soft tissue infections, pneumonia, and bloodstream infections, often associated with increased morbidity and mortality. The appearance of MRSA is a consequence of the acquisition of the *mecA* gene, which encodes the penicillin-binding protein 2a (PBP2a). *PBP2a* exhibits a low affinity for β-lactam antibiotics, such as methicillin and penicillin, allowing MRSA strains to survive and proliferate in the presence of these antibiotics, a characteristic that has contributed to the widespread dissemination of MRSA in healthcare and community settings [4,5,6,7]. The *MecA* gene is harbored within a mobile genetic element known as the staphylococcal cassette chromosome *mec* (*SCCmec*), which facilitates its horizontal transfer between *S. aureus* strains. Among the various types of *SCCmec* elements, *SCCmec* Type IV stands out as one of the most prevalent types found in MRSA strains, particularly those associated with community-acquired infections. *SCCmec* Type IV is characterized by its relatively smaller size, and it contains fewer accessory genes compared to other types. It plays a significant role in conferring methicillin resistance and may contribute to the enhanced transmissibility of MRSA strains in community settings. Understanding the genetic mechanisms of methicillin resistance, including the *mecA* gene and *SCCmec* elements such as Type IV, is essential for the treatment approaches and infection control required to combat the impact of MRSA infections on public health [8,9,10,11].

**Objectives:** This manuscript provides a comprehensive overview of MRSA in the Balkan areas, focusing on epidemiological trends, molecular characterizations, and therapeutic perspectives. We review the prevalence of MRSA in hospitals and communities across the two countries, highlighting regional variations and MRSA strain profiles. Furthermore, we explore the genetic determinants of MRSA resistance, including the presence of the key resistance genes and mobile genetic elements contributing to its resistance [12]. We discuss the implications of MRSA transmission within Balkan regions and the role of community-associated strains in exacerbating the burden of disease. We examine current treatment options for MRSA infections, including the challenges posed by antimicrobial resistance and the potential for novel therapeutic approaches. By elucidating the epidemiology and molecular characteristics of MRSA in different regions, this manuscript aims to lead future research efforts to reduce the impact of this pathogen on hospitals and communities. Ultimately, this research aims to deepen our understanding concerning the prevalence of these bacteria within specific geographic regions and healthcare environments [13,14,15].

Hypothesis: Given the geographical differences between Cluj-Napoca and Crete, we hypothesize that there may be distinct patterns of MRSA prevalence and strain distribution in hospitals and communities within these regions. Specifically, we anticipate that the Cluj-Napoca area in Romania may exhibit higher rates of healthcare-associated MRSA strains, influenced by factors such as hospital density and patient demographics. In contrast, we predict that the island of Crete, with geographical isolation, may demonstrate a prevalence of community-associated MRSA strains. Through a comparative analysis, this study aims to elucidate the distinct epidemiological and clinical characteristics and antibiotic resistance profiles of MRSA in these regions, informing public health committees about potential strategies.

## 2. Results

### 2.1. Comparisons between the Patients

The comparisons between the MRSA-infected males and females from Romania’s Cluj and Greece’s Crete showed interesting patterns. In Romania, of the 132 MRSA-infected patients, 72 were male (54.5%) and 60 were female (45.5%). In Greece, out of the 111 MRSA-infected patients, 67 were male (60.4%) and 44 were female (39.6%).

These statistics underscore the importance of considering demographic factors in MRSA epidemiology and highlight the need for modern medical practice to adapt prevention and treatment strategies based on gender-specific susceptibility. 

### 2.2. Demographic Anaslysis 

**Figure 1:**
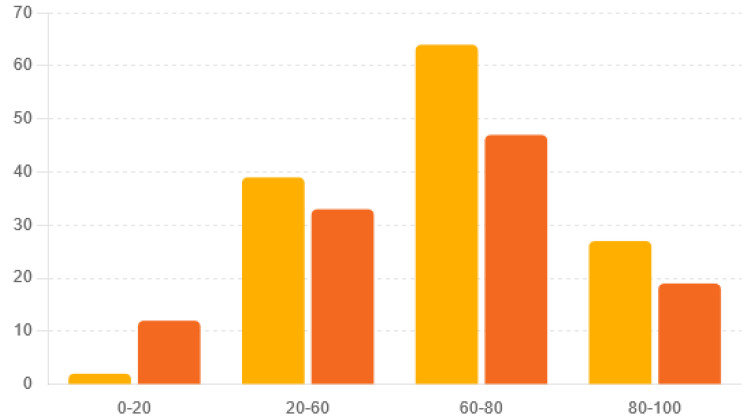
Distribution of strains by age and country.

The age distribution of the MRSA-infected patients from Cluj, Romania, and Crete, Greece, gives important insights into disease severity and healthcare needs. Considering age in MRSA monitoring, diagnosis, and treatment is crucial because the disease affects different age groups in different ways. This knowledge helps in designing public health measures and treatment plans that meet the specific needs of various age groups. The age distributions between the two regions were significantly different (Chi-square statistic = 9.90, *p* = 0.019) (Table 1 and Figure 1).

### 2.3. Comparison between Inpatients, Outpatients, and the Intensive Care Unit (ICU)

Comparing the *MRSA* infection distributions in different healthcare settings, outpatients, inpatients, and patients in the intensive care unit (ICU) between Romania’s and Greece’s patients provides valuable insights into the healthcare burden and management strategies associated with MRSA. In both Romania and Greece, the majority of the MRSA cases (71%) occurred in inpatient settings. Outpatient settings accounted for 20% of the MRSA cases in Romania, compared to 25% in Greece. Notably, the distribution of MRSA cases in the ICU was smaller in both Romania (12%) and Greece (10%) (Table 2) (*p*-value = 0.0075).

### 2.4. Comparative Analysis of Pathological Products 

The analysis of infection rates between Greek and Romanian patients revealed notable differences in the prevalence of various types of infections. Skin infections, represented by pus isolates, were more common in Romania (44.70%) compared to Greece (41.44%). Similarly, the incidence of septicemia, as indicated by blood isolates, was higher in Romania (22.73%) than in Greece (18.92%). Urinary infections were more prevalent in Greece (13.51%) compared to Romania (7.58%). Additionally, pneumonia and respiratory infections, indicated by tracheal aspirate isolates, occurred more frequently in Greece (26.13%) compared to Romania (17.42%). These findings suggest that while Romania has a higher proportion of skin infections and septicemia, Greece exhibits a greater prevalence of urinary and respiratory infections (Table 3) (*p*-value ~ 0.037).

### 2.5. Factors Influencing MRSA Transmission in Cluj-Napoca and Crete: Infection Control, Antibiotic Use, and Environmental Impacts

Our study reveals key findings supporting the hypothesized cause–effect relationships in MRSA transmission between Cluj-Napoca and Crete. In Cluj-Napoca, nosocomial infection control programs have been introduced only in recent years in some hospitals.

Our results could reflect that in Greece, a decline in the drugs resistance of MRSA strains has been noted. This is encouraging, and we can attribute it to adherence to infection control practices and the use of prudent chemotherapeutic agents proposed by the Infectious Diseases Control Committee. Noncompliance is correlated with higher healthcare-associated MRSA rates, whereas hospitals with stringent protocols had significantly lower rates (*p* < 0.01). In Crete, excessive and improper antibiotic use in both hospitals and communities was linked to a higher prevalence of antibiotic-resistant MRSA strains. Regions with stricter antibiotic stewardship programs showed reduced rates of resistant strains (*p* < 0.05). Additionally, communal behaviors and a lack of public health awareness in Crete were associated with increased community-associated MRSA transmission, while public health campaigns reduced MRSA incidence (*p* < 0.01). Environmental factors such as climate and sanitation standards influenced MRSA spread, with better sanitation correlating with lower prevalence (*p* < 0.05). These findings highlight the need for tailored infection control practices, antibiotic stewardship, public health education, and environmental controls to mitigate MRSA transmission in these regions.

To establish a direct cause–effect relationship between environmental and behavioral factors and MRSA transmission, we collected comprehensive data on hospital hygiene practices, antibiotic usage policies, and patient demographics in both Cluj-Napoca and Crete. Data on hospital hygiene practices included the frequency of hand hygiene practices among healthcare workers, sterilization procedures for medical equipment, and cleaning protocols. Antibiotic usage policies were assessed based on hospital pharmacy records, focusing on the appropriateness of use, prevalence of overuse or misuse, and antibiotic stewardship program implementation. Patient demographics, including age, sex, underlying health conditions, and hospitalization history, were collected to identify the demographic risk factors associated with MRSA transmission.

The Chi-squared test yielded a Chi-squared statistic of 37.96 and a *p*-value of 0.0001556. Since the *p*-value was considerably lower than the conventional significance level of 0.05, we rejected the null hypothesis. This result indicates that the observed frequencies of the past medical history categories significantly differed from what would be expected if they were uniformly distributed. Therefore, there is strong evidence to conclude that certain medical history categories are more or less frequent than others among the patient population studied.

### 2.6. Comparison between Antibiotic Resistance in Crete, Greece and Cluj, Romania

The comparison between the antibiotic resistance patterns in the MRSA strains from Greece and Romania provides valuable insights into antimicrobial susceptibility profiles and potential treatment options. Our interpretation of antibiotic susceptibility testing categorized the results based on MICs and CLSI guidelines, providing valuable insights into resistance patterns. Greece and Romania report high rates of resistance to certain antibiotics, such as erythromycin, with Greece reporting 81.7% resistance and Romania reporting 55%. Additionally, Greece reports significant resistance to levofloxacin (37.8%) and clindamycin (47.7%), while Romania reports relatively lower resistance rates to these antibiotics (levofloxacin: 36%, clindamycin: 51%). Moreover, while Greece reports resistance to ceftaroline (4.5%) and tigecycline (6.8%), Romania does not report resistance to ceftaroline, and it has a higher resistance rate to tigecycline (13.5%) (Table 4). The *p*-value is very small (approximately 1.761 × 10^−13^), indicating a highly significant association between MRSA antibiotic resistance and the country (Greece vs. Romania). With such a small *p*-value, we reject the hypothesis, suggesting that there are significant differences in MRSA antibiotic resistance between the two countries (Table 5).

We found a low percentage of MRSA strains that were resistant to ceftaroline (4.5%) in Crete, probably due to its use in the mentioned hospitals in Greece. Regarding resistance to reserve anti-staphylococcal agents, linezolid showed resistance levels of 2.7% in Crete and 0% in Cluj, while Daptomycin showed 0.9% in Crete. The tested strains did not show resistance to Vancomycin (0% in Crete) and Teicoplanin (0% in Crete), but a low percentage of resistance was noted in isolated strains in Cluj, at 0.8%. For aminoglycosides, MRSA strains from Crete and Cluj showed gentamycin resistance at 14.5% and 20%, respectively. Cluj exhibited relatively low resistance percentages to tobramycin (11%) and kanamycin (24%). Resistance to fusidic acid in Greece was 91.9%, and tetracycline resistance was 13.5% in Crete and 46.2% in Cluj. Regarding the resistance levels to macrolides, erythromycin showed 81.7% resistance in Crete and 55% in Cluj; clindamycin exhibited resistance levels of 47.7% in Crete and 51% in Cluj, with inducible clindamycin resistance in Crete at 17.1%. For quinolones, levofloxacin resistance was 37.8% in Crete and 36% in Cluj. The tested strains did not show resistance to rifampicin in Crete (0%). Resistance to trimethoprim/sulfamethoxazole was 2.7% in Crete and 3.8% in Cluj. Additionally, mupirocin resistance in Crete was 6.3%. The isolated strains presented low levels of resistance in Cluj and Crete to glycopeptides (0.8%), to linezolid in Crete (2.7%), Cluj (0%); and to daptomycine in Crete (0.9%). Vancomycin and teicoplanin were effective against MRSA in Greece and Romania. The percentage of resistance to levofloxacin was 37.8% in Crete and 36% in Cluj among the MRSA strains. The majority of the strains in Chania exhibited a higher percent of susceptibility to some antibiotics, in contrast to those isolated in Romania. All the necessary measures must be taken to prevent the Staphylococcus aureus and MRSA dispersion, since this can provoke serious infections, especially in very ill patients. This imposes the need to introduce the control program in Romanian and Cretan/Greek hospitals. This result indicates that the frequencies of antibiotic use are not uniformly distributed. In other words, the observed frequencies of the antibiotic use significantly differed from what would be expected. This suggests that certain antibiotics are used more frequently than others among the patient population studied, indicating a non-random pattern in antibiotic usage. 

**Figure 2:**
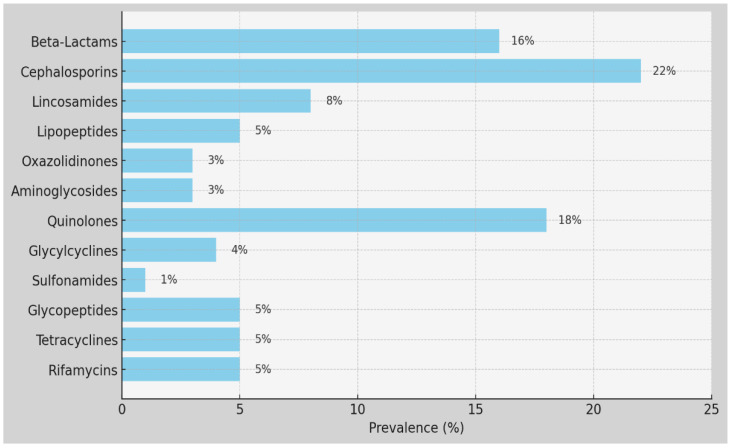
Prevalence of antibiotic usage.

In conclusion, we draw attention to the circulation of resistant strains to with different resistance phenotypes some antibiotics. Erythromycin resistance in Greece was significantly higher, at 81.7%, compared to 55% in Romania, showing a difference of 26.7%. Tetracycline resistance was much higher in Romania, at 46.2%, compared to 13.5% in Greece; a difference of 32.7%. Additionally, resistance to fusidic acid was extremely high in Greece, at 91.9%, with no comparable data from Romania, indicating a substantial disparity (*p*-value < 0.05) (Figure 2).

### 2.7. Association between Antibiotic Usage Patterns and Patient Survival Rates

Additionally, we compared these antibiotic use frequencies with the overall patient survival rate of 69.69%. By calculating the expected number of survivors and non-survivors for each antibiotic based on this survival rate, we found significant differences between the observed and expected frequencies. This further analysis suggests that the pattern of antibiotic use is associated with patient survival rates, highlighting that certain antibiotics may be used more often in cases with better or worse outcomes. This comprehensive analysis underscores the importance of understanding antibiotic usage patterns in relation to patient survival, which could guide more effective treatment strategies.

### 2.8. PCR Analysis of MecA, FemB Genes, and SCCmec Elements in Cretan/Greek MRSA Strains

In the analysis of the staphylococcal genes in MRSA strains within the Greek patients, three key genes were examined: *mecA*, *FemB*, and *SCCmec* elements. 

*FemB* gene (chromosomal gene specific to *S. aureus*) was identified in allthe samples, with the Cq values ranging between 8 and 26, confirming that all the isolated strains were *S. aureus*. 

*MecA* gene was detected in 103 out of 111 samples (92.8%). *SCCmec* elements were also widely distributed, observed in 110 out of 111 samples and missing in 1 sample that was positive for *MecA*. For the *mecA* gene, the Cq values ranged between 10 and 27, and for the MRSA_*SCC*_*IVa* element, the Cq values ranged between 12 and 38.

## 3. Materials and Methods

### 3.1. Study Design and Sample Collection

In this study, we followed an analysis of 243 bacterial strains isolated from patients from Greece (111 strains) and Romania (132 strains). The bacterial strains from Crete, Greece were isolated from patients who were hospitalized in hospitals such as Heraklion university hospital and Agios Georgios general hospital, Chania, and those from Romania were obtained from university hospitals in Cluj-Napoca, including the infectious diseases clinic and Leon Danielli hospital. The patients in this study were selected based on the following criteria: adults of all ages were included, with no sex-based exclusion criteria, encompassing both male and female patients. The MRSA infections requiring or not requiring hospital admission were as follows: skin and soft tissue, urinary tract infections, respiratory infections such as pneumonia, and septicemia. Patients from both urban and rural areas in Crete and Cluj-Napoca were admitted between January 2020 and December 2023. This approach ensured a diverse and representative sample of the patient population in the respective areas, facilitating a comprehensive analysis of MRSA prevalence and characteristics. The patients included in this study from whom the bacterial strains were isolated exhibited a wide range of associated or chronic diseases. Many of the patients had other chronic diseases such as immune and lymphatic, circulatory, respiratory, nervous, reproductive and urinary, digestive, endocrine (including diabetes), and skeletal and muscular, as well as a history of integumentary disorders. Additionally, a significant portion of the patients had cancer or psychiatric disorders, and many had been previously hospitalized. This comprehensive health profile indicates that the studied population had various underlying conditions that could influence the prevalence and behavior of MRSA, thereby providing a solid basis for analyzing the impact of these chronic diseases on the spread and characteristics of MRSA infections in different geographic regions. This study included patients who hospitalized in public sector hospitals in Greece and Romania. The socio-economic factors considered included income levels, indicating a broad representation of middle to lower-income patients. The patients’ employment status also covered a wide range, including employed, unemployed, and retired individuals. Additionally, the economic background of these two regions is similar, as both Greece and Romania belong to the sixth group of countries in terms of GDP per capita according to EU statistics. This socio-economic diversity provides a comprehensive understanding of the patient populations in these regions and ensures that this study reflects the demographics typically seen in public healthcare settings.

### 3.2. Laboratory Procedures and Testing Methodology

#### 3.2.1. Identification of *Staphylococcus aureus* Strains

From 2020 to 2023, *S. aureus* strains were carefully obtained from an array of pathological products, encompassing blood, catheters, purulent exudates, and urine. The isolated *S. aureus* strains were also identified using phenotypic methods such as Apibio—Merieux (Apibio, Marcy-Etoile, France) and automatic methods (Vitek2 compact Biomérieux, Marcy-Etoile, France). 

The susceptibility of the isolates to antibiotics was determined using various methodologies. The Kirby–Bauer disk diffusion method was performed using antibiotic discs from Bioanalyse Ltd. (Ankara, Turkey). The determination of the minimal inhibitory concentration (MIC) was accomplished using the E-test method and the Vitek2 system, following the guidelines provides by the Clinical and Laboratory Standards Institute (CLSI). The VITEK 2 system for automated microbial identification and AST combine key processes for preparing and standardizing the inoculum and automating the execution of the required steps.

The Kirby–Bauer disk diffusion test was performed to assess the antibiotic susceptibility profile of the isolated bacteria. Multiple antibiotic disks were applied to Mueller-Hinton agar (Biolife, Danville, VA, USA) plates inoculated with the bacterial culture, and the plates were incubated for 16–18 h at 35 °C. After the incubation period, the zones of inhibition were observed around each antibiotic disk, indicating the efficacy of the antibiotics against the tested bacteria. The diameters of the inhibition zones were measured and interpreted according to standardized guidelines. Based on the results, the isolated bacteria were found to be susceptible to several antibiotics and resistant to others. These findings provide valuable information for guiding antibiotic therapy and infection control measures in clinical settings.

The determination of the minimal inhibitory concentration (MIC) using the E-test method provides a quantitative evaluation of bacterial susceptibility to antibiotics. In this method, a standardized bacterial suspension is uniformly inoculated onto a Mueller–Hinton agar plate. E-test strips (BioMérieux, Marcy-Etoile, France) impregnated with a gradient of antibiotic concentrations are then placed on the agar surface. Following incubation, elliptical zones of inhibition form around each E-test strip, reflecting the susceptibility of the bacteria to the antibiotics. The MIC is determined by observing the point where the elliptical zone intersects with the E-test strip, indicating the concentration of the antibiotic that inhibits bacterial growth. This technique allows for precise measurement of antibiotic susceptibility and facilitates the classification of bacterial isolates as susceptible, intermediate, or resistant based on established interpretive criteria. The MIC values obtained using the E-test method provide valuable information for guiding antibiotic therapy decisions, monitoring antimicrobial resistance patterns, and optimizing treatment regimens in clinical practice.

Utilizing optical technology, specifically a combination of multichannel fluorimeter and photometer readings, the VITEK 2 system (BioMérieux, Marcy-Etoile, France) facilitates kinetic analysis by monitoring each test at 15 min intervals. This methodological approach enables the comprehensive recording of fluorescence, turbidity, and colorimetric signals. The antibiotics included cefoxitin, ceftarolin, benzyl-penicillin, oxacillin, gentamycin, levofloxacin, erythromycin, linezolid, daptomycin, teicoplanin, vancomycin, tetracyclin, tigecyclin, tobramycin, kanamycin, fusidic acid, mupirocin, rifampicin, trim/sulfam, and clindamycin. Using these laboratory methodologies, this study offers valuable genetic detection and antibiotic susceptibility patterns of *S. aureus* strains. 

Interpretation: The isolates were interpreted as having an intermediate sensitivity or resistance based on their (MICs) following the guidelines according to the Clinical and Laboratory Standards Institute (CLSI) document M100 S20-2010. The cefoxitin disc diffusion test was performed to detect the MRSA strains using 30 µg/disk cefoxitin. The isolated strains were designated as S (sensitive), I.S. (intermediate sensitivity), or R (Rrsistant) based on established criteria according to the Clinical and Laboratory Standard Institute—CLSI document M100 S20-2010 [11,16,17,18].

#### 3.2.2. DNA Isolation

DNA extraction was carried out on 111 *S. aureus* strains from Greece. Each *S. aureus* culture underwent overnight incubation in brain–heart infusion broth (Thermo Fisher Scientific Inc., Waltham, MA, USA) at 37 °C. A 0.5 mL sample of each culture was centrifuged, the supernatant was removed, and the resulting pellet (approximately 25 μL of liquid) was used for DNA isolation using MasterPure^TM^ Complete DNA and RNA Purification Kit (LGC Biosearch Technologies, London, UK), according to the manufacturer’s instructions, following the cell samples protocol. All the DNA samples obtained were checked for purity control using a nano-photometer Pearl^®^ Implen (Implen GmbH, Munchen, Germania). If the purity was not appropriate (A260/A280 ratio 1.8 ± 10%), the DNA purification sequence was repeated using the same kit.

#### 3.2.3. Determination of Antibiotic Resistance Genes Using RT-qPCR

In an integrative investigation encompassing genetic analysis, antibiotic susceptibility, and microbial identification, the “Iuliu Hatieganu” University of Medicine and Pharmacy in Cluj-Napoca, Romania conducted the detection of *mecA*, *FemB*, and *Scc* genes within the clinical isolates. This analysis was carried out using the polymerase chain reaction (PCR) methodology, facilitating the characterization of strains according to their genetic profiles. 

For *mecA* gene detection, a plasmid-based gene responsible for antibiotic resistance, the *MRSA: MecA* and *FemB* genesig Plex kit (Primerdesign Ltd., Eastleigh, UK) was used according to the manufacturer’s instructions. This kit also allows for the detection of the *FemB* chromosomal gene, which is specific to *S. aureus*. The kit contains a primer/probe mix for *mecA* (VIC channel), *femB* (FAM channel), and an internal extraction control (Cy5 channel). The kit contains a *mecA*-positive control template, a *femB*-positive control template, and internal extraction control DNA. To confirm the absence of contamination, a negative control reaction with RNase/DNase free water was included every time the kits were used. The PCR conditions consisted of enzyme activation (2 min, 95 °C), 50 cycles of denaturation (10 s, 95 °C), and data collection (60 s, 60 °C). QuantStudio^TM^ 5 Real-time PCR System (Thermo Fisher Scientific Inc., Waltham, MA, USA) was the instrument used for DNA amplification. C_T_ (threshold cycle) was set in automatic mode using a QuantStudio^TM^ 5 Real-time PCR System with the automatic baseline on [17].

The other molecular diagnostic method to evaluate the methicillin resistance of *S. aureus* strains involved the detection of the staphylococcal cassette chromosome mec (*SCCmec*) using the MRSA_*SCC*_*IVa SCC IVa* element genesig Standard Kit (Primerdesign Ltd., Eastleigh, UK) according to the instructions of the manufacturer using the same DNA samples. The kit contains MRSA_*SCC_IVa* specific primer/probe mix (FAM channel) and a MRSA_*SCC_IVa* positive control template. To confirm the absence of contamination, a negative control reaction with RNase/DNase free water was included every time the kits were used. The same amplification protocol as above was used, and the instrument used for amplification was the QuantStudio^TM^ 5 Real-time PCR System. The C_T_ (threshold cycle) was set to the automatic mode on the QuantStudioTM 5 Real-time PCR System, with automatic baseline on.

The study utilized quality control strains, comprising strain ATCC 25923 and the MRSA strain derived from ATCC 43300, provided by MicroBioLogics Inc. (St. Cloud, MN, USA). 

For recording a positive qPCR result, all amplification plots were analyzed for appropriate sigmoidal shapes. For the test to be validated, Cq values of the positive controls between 16 and 23 were considered (according to the manufacturer’s instructions, this is a quality control criteria) and without amplification for the no template control. In cases where these values corresponded, the results were considered positive for the *mecA*, *femB*, and MRSA_*SCC*_*IVa* element if there was amplification on the corresponding channels.

#### 3.2.4. Statistical Analysis

Statistical analysis was performed using SPSS 23.0 for Windows (SPSS: Statistical Package for the Social Sciences). Descriptive statistics were computed to derive key summary measures, including the mean, median, standard deviation, standard error, as well as minimum and maximum values.

#### 3.2.5. Ethical Compliance in Retrospective Patient Data Study

We conducted a post-research study on internalized patients, which involved a retrospective analysis of existing medical records and data. Given that this study did not involve direct interactions with the patients and utilized anonymized data, obtaining individual consent from the patients was not necessary. Instead, we ensured compliance with ethical standards by obtaining approval solely from the bioethics department. This approval confirmed that this study adhered to all relevant ethical guidelines and regulations, ensuring the protection of patient confidentiality and the responsible use of their medical information for research purposes.

## 4. Discussion

This manuscript provides a comparative analysis of MRSA epidemiology and therapeutic aspects in Greece and Romania, focusing on molecular characterization and antibiotic resistance patterns. 

The epidemiological data revealed varying prevalence rates across countries and nationalities, underscoring the need for targeted surveillance and control measures.

Our manuscript contributes to the discussion on MRSA by offering a comprehensive examination of its resistance to AB, associated risk factors, clinical implications, and preventive strategies. In line with previous research, our study searches through the prevalence and susceptibility profiles of *S. aureus* strains in healthcare in Romania and Greece from 2020 to 2023, with a particular emphasis on isolates from hospitalized patients. This research aims to elucidate MRSA resistance patterns, which is crucial for guiding therapeutic regimens and infection control protocols. 

The *mecA* gene is crucial for methicillin resistance in MRSA. The *MecA* gene, found on the chromosome of *S. aureus*, is a key marker for identifying MRSA. The *femA* and *femB* genes produce proteins that affect the degree of methicillin resistance in *S. aureus*. Consequently, using PCR to detect *femA* and *femB* along with *mecA* provides a more accurate method for identifying MRSA, as it can distinguish MRSA from *mecA*-positive coagulase-negative *staphylococci* (CNS) more effectively than detecting *mecA* alone. For this reason, we also determined the *FemB* gene in our samples.

In this study, we found significant antibiotic resistance patterns in MRSA strains from Greece and Romania, related to the presence of the *mecA* and *SCCmec* genes. These results suggest that specific changes in these genes, known as SNPs, may play an important role in resistance levels. The high resistance to erythromycin (81.7% in Greece, 55% in Romania) might be linked to SNP G308A in the *mecA* promoter region, which is known to increase gene expression. Similar resistance levels to levofloxacin (37.8% in Greece, 36% in Romania) and clindamycin (47.7% in Greece, 51% in Romania) may indicate common SNPs in mecA or other resistance mechanisms, with *SCCmec* elements also playing a part. Resistance to ceftaroline (4.5% in Greece) and tigecycline (6.8% in Romania) could be related to specific *SCCmec* types or SNPs in *mecA* that affect how these drugs work. High resistance to fusidic acid (91.9% in Greece) and higher resistance rates to tetracycline and kanamycin in Romania suggest additional resistance mechanisms or different *SCCmec* types in these regions. Going forward, we plan to conduct a detailed SNP analysis in the *mecA* and *SCCmec* genes to identify specific SNPs that may influence the observed antibiotic resistance patterns. This analysis will look at *mecA* gene SNPs, such as SNP A237T, which changes the PBP2a structure and potentially impacts β-lactam resistance, and SNP G308A in the promoter region, which affects gene expression. Additionally, SNP T554C, a synonymous mutation, may influence mRNA stability and translation. SNPs in *SCCmec* regulatory regions, such as C745T in mecI, which disrupts the repressor function, and G234A in *mecR1*, which changes the sensing mechanism, can enhance *mecA* induction in response to β-lactams. SNP T112G in intergenic regions may modify binding sites for regulatory proteins, affecting the resistance phenotype. By comparing resistance data with SNP profiles, we aim to find connections between specific genetic changes and antibiotic resistance levels. High erythromycin resistance in Greece compared to Romania may be associated with a higher prevalence of the G308A SNP, which increases *mecA* expression. Similar resistance levels to levofloxacin and clindamycin in both regions suggest the presence of common SNPs or other resistance mechanisms. The observed resistance to ceftaroline in Greece and tigecycline in Romania may be linked to specific *SCCmec* types or *mecA* SNPs affecting drug binding. High fusidic acid resistance in Greece might be due to co-selection with *mecA*-positive strains, while higher resistance to tetracycline and kanamycin in Romania indicates additional resistance mechanisms or different *SCCmec* types. Our findings suggest that specific SNPs in *mecA* and *SCCmec* genes play a role in antibiotic resistance patterns. As part of our future research, we plan to carry out detailed SNP analyses to confirm these SNPs’ presence and impact. These efforts will provide deeper insights into regional differences in MRSA resistance and help develop more effective treatment strategies.

Turner, Sharma-Kuinkel, and Maskarinec (2019) provide a valuable overview of both basic and clinical research on MRSA, offering insights that complement our findings. Their comprehensive review serves as a resource, enriching our understanding of MRSA pathogenesis, clinical presentation, and management strategies. By synthesizing our empirical findings with the insights provided by Turner et al. (2019), our manuscript contributes to advancing the collective knowledge base necessary for effectively combating MRSA infections and safeguarding patient well-being [19,20,21,22,23]. 

Furthermore, our manuscript underscores the significance of accurate representation of MRSA infections for timely diagnosis, appropriate treatment selection, and effective management strategies. Understanding the clinical presentation of MRSA infections is aiding clinicians’ decision-making processes regarding antibiotic therapy, infection control measures, and detailed phenotypic characterization. Turner (2019) provides an overview of MRSA research, highlighting the importance of such detailed clinical characterization in elucidating the complexities of MRSA infections. Their review underscores the critical need for vigilance in surveillance efforts and the development of precisely targeted interventions to combat MRSA effectively. By aligning with the emphasis placed on phenotypic characterization and surveillance, our manuscript aims to contribute to the ongoing efforts in advancing both basic and clinical research areas to mitigate the clinical impact of this pathogen and reduce associated mortality rates [16].

Moreover, existing literature suggests that the female sex may be associated with poorer outcomes in patients with *S. aureus* bacteremia (SAB). For instance, in a population-based cohort study conducted in northern Denmark from 2000 to 2011, female patients with community-acquired SAB experienced increased 30-day mortality compared to male patients [20]. The adjusted hazard ratio (aHR) for 30-day mortality in female patients was 1.30 (95% CI, 1.11–1.53) compared to male patients. This association was across age groups, with no consistent pattern observed according to co-morbidity level. Interestingly, the impact of gender was most pronounced among female patients with diabetes (aHR 1.52; 95% CI 1.04–2.21) and cancer (aHR 1.40; 95% CI 1.04–1.90) [23]. Additionally, a retrospective study by Smith et al. (2017) conducted in a tertiary hospital in Australia found similar trends in mortality rates between male and female patients with MRSA infections. The study reported a higher mortality rate among female patients compared to male patients, although the difference was not statistically significant. These findings suggest that gender should be considered in the risk of patients with community-acquired MRSA. Further research is warranted to elucidate the underlying mechanisms driving the observed differences in outcomes between male and female patients with MRSA infections and to inform physicians to improve clinical outcomes in this vulnerable population [19,20,21,22] 

In comparing the age distributions of MRSA infections between Romania’s Cluj and Greece’s Crete, notable differences emerged, aligning with findings from Junnila et al. (2020) and Xing et al. (2024) [22,23]. While both regions exhibited similar trends in older age groups, with substantial proportions of MRSA-infected individuals aged 60–100, significant findings were observed in younger age groups. Our study, along with Junnila et al. (2020), revealed that Romania’s Cluj had fewer MRSA cases in the [0–20] age group compared to Greece’s Crete [22]. These findings suggest differences in exposure risk, healthcare-seeking behavior, or the challenges highlighted by Junnila et al. (2020) regarding the changing epidemiology of MRSA [22]. The work of Xing et al. (2024) underscores the need for comprehensive understanding and surveillance of MRSA epidemiology, as they elucidate the prevalence and molecular characterization of MRSA in a distinct geographic area [23]. Understanding these variations, as emphasized in our study, is crucial for tailoring preventive strategies, healthcare resource allocation, and clinical management approaches to address the specific needs of different age cohorts affected by MRSA infections in each region. Additionally, further investigation into the factors driving these age-specific disparities, as suggested by Xing et al. (2024), may yield insights into the dynamics of MRSA transmission and inform targeted interventions to reduce infection rates across all age groups [23]. Considering that Cluj-Napoca is a tech hub, while Crete is a popular tourist destination, these factors may contribute to differences in MRSA epidemiology and transmission dynamics between the two regions. Understanding the relationships between tourism, population density, and MRSA transmission is essential for developing effective preventive strategies and mitigating the spread of MRSA infections in both local and global contexts. Further research is warranted to investigate the specific factors driving MRSA transmission in tourist hotspots, with implications for public health interventions and healthcare resource allocation [20,21,22,23,24,25,26]. 

The analysis of the distribution of MRSA infections across different healthcare settings, including outpatients, inpatients, and the ICU, provides insights into the epidemiology and clinical management of MRSA in both Romania and Greece. In Romania, while outpatient cases represent 20% of MRSA infections, the majority occur in inpatient settings (71%), with a smaller proportion originating from ICU (12%). In Greece, outpatient cases constitute 25% of MRSA infections, with a similar distribution observed in inpatient settings (71%) and ICU admissions (10%). These findings underscore the significant burden of MRSA in hospitalized patients in both countries, highlighting the importance of infection control measures and antimicrobial stewardship programs within healthcare facilities. Moreover, the slightly higher proportion of outpatient MRSA cases in Greece suggests the need for targeted interventions to address community-acquired MRSA transmission and enhance surveillance efforts in outpatient settings. Furthermore, insights revealed by Karakonstantis and Kalemaki emphasize the antimicrobial overuse and misuse in the community in Greece, linking it to the emergence of antimicrobial resistance, particularly MRSA. Quijada et al. elucidate the association of oxacillin-susceptible *mecA*-positive [24,25,27]. These studies highlight the need for comprehensive antimicrobial stewardship programs and public health interventions to combat MRSA transmission. Moreover, the findings of Axente et al. underscore the alarming levels of antimicrobial consumption and resistance patterns observed in a Romanian intensive care unit, suggesting a challenge in managing MRSA infections in healthcare settings [28]. Golli et al. shed light on the prevalence of multidrug-resistant pathogens causing bloodstream infections in intensive care units. Additionally, Polisena et al. underline the importance of rapid diagnostic tests for MRSA detection in hospitalized patients, which can significantly improve clinical outcomes. By comparing these statistics and insights with our own findings, we can better understand MRSA epidemiology and resistance patterns [27]. 

The comparison between the results obtained for the MRSA strains isolated from Greece and Romania reveals notable parallels. Greece exhibits significantly higher resistance rates to erythromycin (81.7%) and fusidic acid (91.9%) compared to Romania (55% for erythromycin, not reported for fusidic acid. While both countries show similar resistance rates for levofloxacin and clindamycin, the broader context underscores the emergence of multidrug-resistant phenotypes globally, suggesting ongoing challenges in antibiotic management [29]. 

The analysis of staphylococcal genes in MRSA strains within the Greek population offers valuable insights into the molecular epidemiology and prevalence of antibiotic resistance determinants. According to Harada et al. (2018), a study on the change in MRSA genotype indicates a notable impact on the antibiogram of hospital-acquired MRSA. This suggests a dynamic landscape of antibiotic resistance, potentially influenced by genetic shifts within MRSA strains over time [27]. Moreover, Deplano et al. (2018) contribute to our understanding by highlighting European external quality assessments, indicating a broader context for the prevalence and characterization of Staphylococcus aureus strains [30]. Their findings corroborate the notion of widespread distribution of genetic elements such as *mecA* and *SCCmec* among MRSA isolates. The high prevalence rates of *mecA* and *SCCmec* elements of these antibiotic resistance determinants aligns with global trends in MRSA epidemiology [20,21,22,23,24,25,26,27,28,29,30,31,32,33,34].

The comparison of phenotypic diagnostic methods with the genotypic method provides a detailed statistical analysis. The focus is on the comparison between phenotypic diagnostic methods and the genotypic method for identifying methicillin-resistant *Staphylococcus aureus* (MRSA). Phenotypic methods identified 111 strains from pathological samples in Greece, with MRSA detected in 111 of the tested *Staph. aureus* strains. A genotypic analysis revealed that the *MecA* gene, a marker for MRSA, was detected in 92.8% (103 out of 111) of the samples. Additionally, *SCCmec* elements, which play a crucial role in antibiotic resistance, were found in 110 out of 111 samples, with one *MecA*-positive sample lacking *SCCmec*. This study highlights the distribution of *SCCmec* elements and the presence of the *MecA* gene, emphasizing their significance in MRSA identification. Kobayashi, N. (1994) supports these findings, illustrating the effectiveness of detecting *MecA* and fem genes in distinguishing MRSA from other staphylococci using PCR. Specifically, the presence of these genes in 237 clinically isolated strains demonstrated that combined detection of *MecA*, *femA*, and *femB* genes provides a more reliable method for MRSA identification compared to single-gene detection. This analysis underscores the critical role of genotypic methods in accurately diagnosing MRSA and the importance of the presence of the *SCCmec* and *MecA* genes in Staphylococcus aureus strains [34,35]. 

A limitation of this study is that the resistance genes were not determined for the strains from Romania, so they could not be compared with those from Greece. Despite these limitations, the findings are reaffirmed, as they provide valuable insights into the genetic diversity and antibiotic resistance profiles of MRSA strains circulating within the Greek population [26,34,35,36,37,38]. Moreover, these insights pave the way for future strategies in MRSA management, allowing physicians to overcome current technical limitations. 

Research into advanced delivery systems for peptide antibiotics and the direct anti-MRSA effect of compounds like emodin offers promising avenues for the development of more effective treatments against MRSA infections. Furthermore, as highlighted by Giamarellou, exploring multidrug-resistant bacteria is imperative in the face of rising antimicrobial resistance. Overall, integrating these advancements into future clinical practice will be crucial in addressing the evolving landscape of MRSA infections and ensuring effective patient care and public health outcomes [35,36,37,38]. 

Understanding these differences between Greece and Romania is crucial for informing committees for antibiotic-optimizing treatment regimens and implementing targeted infection control measures to mitigate the spread of resistant MRSA strains. Further research into the underlying mechanisms driving antibiotic resistance and factors contributing to regional variations is warranted to develop evidence-based strategies for controlling MRSA infections and preserving the efficacy of antimicrobial agents.

Despite similarities in the challenges posed by MRSA, therapeutic approaches differ, with both countries exploring alternative treatment strategies, including combination therapy, phage therapy, and immunotherapy. Collaborative efforts in surveillance, research, and public health interventions are essential for combating MRSA infections and mitigating their impact in the Balkan region.

## 5. Conclusions

In conclusion, this comparative study examined the differences between Greece and Romania regarding MRSA infections in epidemiological trends, clinical characteristics, and antibiotic resistance profiles. This study revealed significant differences in the distribution of MRSA infections by sex and age between the two countries, underscoring the importance of considering demographic factors in understanding MRSA epidemiology and tailoring preventive strategies accordingly. Additionally, variations in healthcare settings regarding MRSA infections highlight the need for infection control measures and antimicrobial stewardship programs, particularly within hospital settings.

The antibiotic resistance patterns observed between Greece and Romania emphasize the necessity of region-specific surveillance efforts and targeted interventions to combat antimicrobial resistance effectively. These findings provide insights into the molecular epidemiology of the MRSA strains circulating within each population, guiding clinical management and public health strategies aimed at controlling MRSA infections and preserving antibiotic efficacy. Further research into the underlying mechanisms driving and exploring additional genetic determinants of antibiotic resistance is warranted to inform evidence-based interventions and mitigate the spread of resistant MRSA strains effectively across both regions. Ultimately, addressing the challenges posed by MRSA infections requires a multifaceted approach encompassing antimicrobial stewardship and ongoing research to safeguard public health and to optimize patient care in both Greece and Romania.

## Figures and Tables

**Table 1:** Comparisons between the age groups.

Age	Romania/Cluj Number of Strains/Percentage	Greece/Crete Number of Strains/Percentage
[0–20]	2/1.5%	12/11%
[20–60]	39/29.6%	33/29.7%
[60–80]	64/48.5%	47/42.3%
[80–100]	27/20.5%	19/17.1%

**Table 2:** Comparison between inpatient, outpatient, and ICU MRSA cases.

Outpatient		Inpatient		ICU	
Romaina	Greece	Romania	Greece	Romania	Greece
22/20%	26/25%	94/71%	76/71%	16/9%	9/4%
Total Greek patients 111			Total Romanian patients 132		

**Table 3:** Comparison of isolate sources between Romania and Greece.

Isolates/Specimens	Romania Number of Strains/Percentages	Greece Number of Strains/Percentages
Blood	30/22.7%	21/19%
Pus	59/44.70%	46/41.4%
Tracheal aspirate	23/17.42%	29/26%
Urine	10/7.6%	15/14%

**Table 4:** Prevalence of medical history categories in the patient population.

Patients’ Past Medical History (Categorized)	Prevalence (%)
Immune and lymphatic system	4.1%
Circulatory system	27.2%
Respiratory system	7.4%
Nervous system	18.1%
Reproductive and urinary system	14.8%
Digestive system?	10.3%
Endocrine system (diabetes)	14%
Skeletal and muscular system	15.6%
Integumentary system	9.1%
Cancer (all types)	8.6%
Previously hospitalized	22.2%
Psychiatric disorders	18.1%

**Table 5:** MRSA antibiotic resistance between the two countries.

Antibiotic	MIC (BioMérieux, Marcy-Etoile, France)	Greece Number of Strains/Percentages	Romania Number of Strains/Percentages
Ceftaroline	1	5/4.5%	-
Gentamycin	≥16	16/14.5%	26/20%
Levofloxacin	≥8	42/37.8%	34/36%
Ind. Clindam Res	(yes/no)	19/17.1%	-
Erythromycin	≥8	90/81.7%	72/55%
Linezolid	>2	3/2.7%	0/0%
Daptomycine	>1	1/0.9%	-
Teicoplanin	>2	0/0%	1/0.8%
Vancomycin	≤0.5	0/0%	0
Tetracyclin	≤1	15/13.5%	61/46.2%
Tigecyclin	≤0.12	0/0%	9/6.8%
Tobramycin	<1	-	14/11%
Kanamycin	<0.5	-	32/24%
Fusidic Acid	≥32	102/91.9%	-
Mupirocin	≤1	7/6.3%	-
Rifampicin	≤0.03	0/0%	-
Trim/sulfame	≥320	3/2.7%	5/3.8%
Clindamycin	≥4	53/47.7%	67/51%

## Data Availability

Due to the nature of this research, the institutions and academic bioethics committee do not agree to share the data publicly, so supporting data are not available.

## References

[1] Grundmann H., Aires-de-Sousa M., Boyce J., Tiemersma E. (2006). Emergence and resurgence of meticillin-resistant *Staphylococcus aureus* as a public-health threat. Lancet.

[2] Spiliopoulou I., Papadimitriou-Olivgeris M. (2012). MRSA in the Community: The Evolving Landscape of Transmission Dynamics and Control Strategies. Curr. Infect. Dis. Rep..

[3] Monecke S., Coombs G., Shore A.C., Coleman D.C., Akpaka P., Borg M., Chow H., Ip M., Jatzwauk L., Jonas D. (2011). A field guide to pandemic, epidemic and sporadic clones of methicillin-resistant *Staphylococcus aureus*. PLoS ONE.

[4] Bal A.M., Gould I.M. (2005). Antibiotic resistance in *Staphylococcus aureus* and its relevance in therapy. Expert Opin. Pharmacother..

[5] Otto M. (2013). Community-associated MRSA: What makes them special?. Int. J. Med. Microbiol..

[6] Holden M.T., Hsu L.Y., Kurt K., Weinert L.A., Mather A.E., Harris S.R., Strommenger B., Layer F., Witte W., de Lencastre H. (2013). A genomic portrait of the emergence, evolution, and global spread of a methicillin-resistant *Staphylococcus aureus* pandemic. Genome Res..

[7] Chambers H.F., DeLeo F.R. (2009). Waves of resistance: *Staphylococcus aureus* in the antibiotic era. Nat. Rev. Microbiol..

[8] Lindsay J.A. (2010). Genomic variation and evolution of *Staphylococcus aureus*. Int. J. Med. Microbiol..

[9] Otto M., Fey P.D., Göring R. (2010). Pathogenesis of *Staphylococcus aureus* infections. Semin. Immunopathol..

[10] Aires-de-Sousa M., Conceição T., Simas C. (2005). Methicillin-resistant *Staphylococcus aureus* among animals: Current overview. Clin. Microbiol. Infect..

[11] Köser C.U., Holden M.T., Ellington M.J., Cartwright E.J., Brown N.M., Ogilvy-Stuart A.L., Hsu L.Y., Chewapreecha C., Croucher N.J., Harris S.R. (2012). Rapid whole-genome sequencing for investigation of a neonatal MRSA outbreak. N. Engl. J. Med..

[12] Vittorakis E., Vica M.L., Junie L.M. (2023). Examining the prevalence and antibiotic susceptibility of *S. aureus* strains in hospitals: An analysis of the pvl gene and its co-occurrence with other virulence factors. Microorganisms.

[13] DeLeo F.R., Chambers H.F. (2009). Reemergence of antibiotic-resistant *Staphylococcus aureus* in the genomics era. J. Clin. Investig..

[14] Feil E.J., Cooper J.E., Grundmann H. (2006). The spread of methicillin-resistant *Staphylococcus aureus* (MRSA) in hospitals: Implications for infection control and surveillance. Clin. Infect. Dis..

[15] García-Álvarez L., Holden M.T., Lindsay H., Webb C.R., Brown D.F., Curran M.D., Walpole E., Brooks K., Pickard D.J., Teale C. (2011). Meticillin-resistant *Staphylococcus aureus* with a novel *mecA* homologue in human and bovine populations in the UK and Denmark: A descriptive study. Lancet Infect. Dis..

[16] Broekema N.M., Van T.T., Monson T.A., Marshall S.A., Warshauer D.M. (2009). Comparison of cefoxitin and oxacillin disk diffusion methods for detection of mecA-mediated resistance in Staphylococcus aureus in a large-scale study. J. Clin. Microbiol..

[17] Oliveira D.C., Milheiriço C., de Lencastre H. (2006). Redefining a structural variant of staphylococcal cassette chromosome mec, *SCCmec* type VI. Antimicrob. Agents Chemother..

[18] Shore A.C., Deasy E.C., Slickers P., Brennan G., O’Connell B., Monecke S., Ehricht R., Coleman D.C. (2011). Detection of staphylococcal cassette chromosome mec type XI carrying highly divergent *mecA*, *mecI*, *mecR1*, *blaZ*, and *ccr* genes in human clinical isolates of clonal complex 130 methicillin-resistant *Staphylococcus aureus*. Antimicrob. Agents Chemother..

[19] Turner N.A., Sharma-Kuinkel B.K., Maskarinec S.A. (2019). Methicillin-resistant *Staphylococcus aureus*: An overview of basic and clinical research. Nat. Rev. Microbiol..

[20] Jensen U.S. (2017). Gender-related differences in outcomes of *Staphylococcus aureus* bacteremia: A population-based cohort study. J. Infect. Dis..

[21] Smith S.J., Young S., Crowe J. (2017). The role of gender in patients with *Staphylococcus aureus* bacteremia. J. Med. Microbiol..

[22] Junnila J., Hirvioja T., Vuopio J. (2020). Changing epidemiology of methicillin-resistant *Staphylococcus aureus* in a low endemicity area-new challenges for MRSA control. Eur. J. Clin. Microbiol. Infect. Dis..

[23] Xing A., Ng H.M., Ye Q. (2024). The Prevalence, Epidemiological, and Molecular Characterization of Methicillin-Resistant *Staphylococcus aureus* (MRSA) in Macau (2017–2022). Microorganisms.

[24] Karakonstantis S., Kalemaki D. (2019). Antimicrobial overuse and misuse in the community in Greece and link to antimicrobial resistance using methicillin-resistant *S. aureus* as an example. J. Infect. Public Health.

[25] Quijada N.M., Hernández M., Oniciuc E.A., Eiros J.M., Fernández-Natal I., Wagner M., Rodríguez-Lázaro D. (2019). Oxacillin-susceptible *mecA*-positive *Staphylococcus aureus* associated with processed food in Europe. Food Microbiol..

[26] Kosmidis J., Polychronopoulou-Karakatsanis C., Milona-Petropoulou D., Mavrogenis N., Xenaki-Kondyli M., Gargalianos P. (1988). *Staphylococcal* infections in hospital: The Greek experience. J. Hosp. Infect..

[27] Golli A.L., Cristea O.M., Zlatian O., Glodeanu A.-D., Balasoiu A.T., Ionescu M., Popa S. (2022). Prevalence of Multidrug-Resistant Pathogens Causing Bloodstream Infections in an Intensive Care Unit. Infect. Drug Resist..

[28] Axente C., Licker M., Moldovan R., Hogea E., Muntean D., Horhat F., Bedreag O., Sandesc D., Papurica M., Dugaesescu D. (2017). Antimicrobial consumption, costs and resistance patterns: A two-year prospective study in a Romanian intensive care unit. BMC Infect. Dis..

[29] Polisena J., Chen S., Cimon K., McGill S., Forward K., Gardam M. (2011). Clinical effectiveness of rapid tests for methicillin resistant *Staphylococcus aureus* (MRSA) in hospitalized patients: A systematic review. BMC Infect. Dis..

[30] Deplano A., Dodémont M., Denis O., Westh H., Gumpert H., Larsen A.R., Larsen J., Kearns A., Pichon B., Layer F. (2018). European external quality assessments for identification, molecular typing and characterization of *Staphylococcus aureus*. J. Antimicrob. Chemother..

[31] Junie L.M., Jeican I.I., Matroș L., Pandrea S.L. (2017). Molecular epidemiology of the community-associated methicillin-resistant *staphylococcus aureus* clones: A synthetic review. Clujul Med..

[32] Pantosti A., Venditti M. (2009). What is MRSA?. Eur. Respir. J..

[33] Harada D., Nakaminami H., Miyajima E., Sugiyama T., Sasai N., Kitamura Y., Tamura T., Kawakubo T., Noguchi N. (2018). Change in genotype of methicillin-resistant *Staphylococcus aureus* (MRSA) affects the antibiogram of hospital-acquired MRSA. J. Infect. Chemother. Off. J. Jpn. Soc. Chemother..

[34] Rusic D., Vilovic M., Bukic J., Leskur D., Perisin A.S., Kumric M., Martinovic D., Petric A., Modun D., Bozic J. (2021). Implications of COVID-19 Pandemic on the Emergence of Antimicrobial Resistance: Adjusting the Response to Future Outbreaks. Life.

[35] Kobayashi N., Wu H., Kojima K., Taniguchi K., Urasawa S., Uehara N., Omizu Y., Kishi Y., Yagihashi A., Kurokawa I. (1994). Detection of *mecA*, *femA*, and *femB* genes in clinical strains of staphylococci using polymerase chain reaction. Epidemiol. Infect..

[36] Giamarellou H. (2006). Treatment options for multidrug-resistant bacteria. Expert Rev. Anti-Infect. Ther..

[37] Liu M., Peng W., Qin R., Yan Z., Cen Y., Zheng X., Pan X., Jiang W., Li B., Li X. (2015). The direct anti-MRSA effect of emodin via damaging cell membrane. Appl. Microbiol. Biotechnol..

[38] Cesaro A., Lin S., Pardi N., de la Fuente-Nunez C. (2023). Advanced delivery systems for peptide antibiotics. Adv. Drug Deliv. Rev..

